# Molecular surveillance of potential SARS-CoV-2 reservoir hosts in wildlife rehabilitation centers

**DOI:** 10.1080/01652176.2023.2164909

**Published:** 2023-01-18

**Authors:** Juan Mena, Christian Hidalgo, Daniela Estay-Olea, Nicole Sallaberry-Pincheira, Antonella Bacigalupo, André V. Rubio, Diego Peñaloza, Carolina Sánchez, Javiera Gómez-Adaros, Valeria Olmos, Javier Cabello, Kendra Ivelic, María José Abarca, Diego Ramírez-Álvarez, Marisol Torregrosa Rocabado, Natalia Durán Castro, Martina Carreño, Gabriela Gómez, Pedro E. Cattan, Galia Ramírez-Toloza, Sofía Robbiano, Carla Marchese, Eduardo Raffo, Paulina Stowhas, Gonzalo Medina-Vogel, Carlos Landaeta-Aqueveque, René Ortega, Etienne Waleckx, Daniel Gónzalez-Acuña, Gemma Rojo

**Affiliations:** a Instituto de Ciencias Agroalimentarias, Animales y Ambientales (ICA3), Universidad de O'Higgins, San Fernando, Chile; b Núcleo de Investigaciones Aplicadas en Ciencias Veterinarias y Agronómicas (NIAVA), Universidad de Las Américas, Chile; c Unidad de Rehabilitación de Fauna Silvestre (UFAS), Escuela de Medicina Veterinaria, Universidad Andres Bello, Santiago, Chile; d Institute of Biodiversity, Animal Health and Comparative Medicine, University of Glasgow, Glasgow, United Kingdom; e Departamento de Ciencias Biológicas Animales, Facultad de Ciencias Veterinarias y Pecuarias, Universidad de Chile, Santiago, Chile; f Departamento de Áreas Silvestres Protegidas, Corporación Nacional Forestal (CONAF), Región del Libertador General Bernardo O’Higgins, Rancagua, Chile; g Centro de Rehabilitación y Exhibición de Fauna Silvestre, Rancagua, Chile; h Centro de Conservación de la Biodiversidad, Ancud, Chile; i Refugio Animal Cascada, Centro de Rehabilitación y Exhibición de fauna nativa de la Fundación Acción Fauna, Santiago, Chile; j Comité Nacional Pro Defensa de la Fauna y Flora (CODEFF), Santiago, Chile; k Servicio Agrícola y Ganadero de Chile (SAG), Unidad de Vida Silvestre, Rancagua, Chile; l Médico Veterinaria Encargada Sección Salud Animal, Zoológico Nacional del Parque Metropolitano, Santiago, Chile; m Médico Veterinaria Sección Salud Animal, Zoológico Nacional del Parque Metropolitano, Santiago, Chile; n Huellitas por un Sueño, Isla de Maipo, Chile; o Departamento de Áreas Silvestres Protegidas, Corporación Nacional Forestal (CONAF), Región de Aysén, Chile; p Departamento de Medicina Preventiva, Facultad de Ciencias Veterinarias y Pecuarias, Universidad de Chile, Santiago, Chile; q Centro de Rehabilitación de Fauna Silvestre, Facultad de Ciencias Veterinarias, Universidad de Concepción, Chillán, Chile; r Servicio Agrícola y Ganadero de Chile (SAG), Unidad de Vida Silvestre, Valdivia, Chile; s Programa Nacional Integrado de Gestión de Especies Exóticas Invasoras, Ministerio del Medio Ambiente, Santiago, Chile; t Centro de Investigación para la Sustentabilidad (CIS), Universidad Andres Bello, Santiago, Chile; u Departamento Patología y Medicina Preventiva, Facultad de Ciencias Veterinarias, Universidad de Concepción, Chillán, Chile; v Institut de Recherche pour le Développement, UMR INTERTRYP IRD, CIRAD, Université de Montpellier, Montpellier, France; w Laboratorio de Parasitología, Centro de Investigaciones Regionales “Dr Hideyo Noguchi”, Universidad Autónoma de Yucatán, Mérida, México; x Departamento Ciencia Animal, Facultad de Ciencias Veterinarias, Universidad de Concepción, Chillán, Chile

**Keywords:** Chile, wildlife conservation, wildlife rehabilitation centers, COVID-19, SARS-CoV-2

## Abstract

**Background:**

The COVID-19 pandemic, caused by SARS-CoV-2 infection, has become the most devastating zoonotic event in recent times, with negative impacts on both human and animal welfare as well as on the global economy. Although SARS-CoV-2 is considered a human virus, it likely emerged from animals, and it can infect both domestic and wild animals. This constitutes a risk for human and animal health including wildlife with evidence of SARS-CoV-2 horizontal transmission back and forth between humans and wild animals.

**Aim:**

Molecular surveillance in different wildlife rehabilitation centers and wildlife associated institutions in Chile, which are critical points of animal-human interaction and wildlife conservation, especially since the aim of wildlife rehabilitation centers is to reintroduce animals to their original habitat.

**Materials and Methods:**

The survey was conducted in six WRCs and three wildlife associated institutions. A total of 185 samples were obtained from 83 individuals belonging to 15 different species, including vulnerable and endangered species. Each specimen was sampled with two different swabs: one oropharyngeal or nasopharyngeal according to the nostril diameter, and/or a second rectal sample. RNA was extracted from the samples and two different molecular assays were performed: first, a conventional RT-PCR with pan-coronavirus primers and a second SARS-CoV-2 qPCR targeting the N and S genes.

**Results:**

All 185 samples were negative for SARS-CoV-2.

**Clinical relevance:**

This study constitutes the first report on the surveillance of SARS-CoV-2 from wildlife treated in rehabilitation centers in Chile, and supports the biosafety procedures adopted in those centers.

## 1. Introduction

The current COVID-19 pandemic, caused by the coronavirus SARS-CoV-2, has infected more than 250 million humans and has caused more than 5 million deaths worldwide (WHO 2022). Early in the pandemic, based on the ability of coronaviruses to infect different vertebrate hosts (Kayode et al. 2021), many species were proposed as the zoonotic origin of SARS-CoV-2 (Gupta et al. 2021; Islam et al. 2022; Shahhosseini et al. 2021; K. Sharun et al. 2021a). Bats were presented as the natural reservoir of SARS-CoV-2, since chiropterans are the natural reservoir hosts of SARS and MERS, among other zoonotic viruses (Letko et al. 2020; Alves et al. 2021; Gupta et al. 2021; Hernandez-Aguilar et al. 2021; Jacob Machado et al. 2021; Kayode et al. 2021; Ruiz-Aravena et al. 2022). Because there were no SARS-like viruses obtained from bats that perfectly matched the sequence of SARS-CoV-2, an unknown intermediate host was proposed as the bridge before it became a human infection (Farrag et al. 2021). Although the spillover model was accurate for MERS (Gupta et al. 2021; Jacob Machado et al. 2021; Weidinger et al. 2021), currently there is no experimental data proving the spillover model for SARS and SARS-CoV-2 infections (Frutos et al. 2021; Jacob Machado et al. 2021). Nonetheless, wildlife can play a critical role in the transmission of SARS-CoV-2, as was the case with minks (*Neovison vison*). SARS-CoV-2 infection in mink farms showed horizontal transmission of the virus from humans to mink, between minks, and from minks to humans (Rabalski et al. 2021; Shriner et al. 2021). These transmission events caused the emergence of a new variant named “Cluster 5”, which had a higher affinity to the ACE2 receptor (Peacock et al. 2021; K. Sharun et al. 2021a), leading to a massive culling of minks in different countries (Frutos et al. 2021). Likewise, white-tailed deer (*Odocoileus virginianus*) in North America display a high rate of infection, with hundreds of cases reported (Le Page 2021; Kuchipudi et al. 2022). Therefore, the risk of SARS-CoV-2 infection in wildlife is relevant both for wild animal health and, to an extent, ecosystem health, as well as human health, since wildlife can act as reservoirs of many infectious diseases (Grange et al. 2021).

A suitable host for a virus, has target cells available to become infected and allows efficient replication, so it can then spread to other individuals (Frutos et al. 2021). In the case of SARS-CoV-2, the infection of target cells occurs through recognition of the angiotensin-converting enzyme 2 (ACE2) receptor by the spike protein of the virus (Devaux et al. 2020). Many *in silico* analyses quickly identified species that were more or less susceptible to SARS-CoV-2 infection based on the receptor binding domain (RBD) of the spike protein and ACE2 receptor coding sequences (Islam et al. 2022). Based on ACE2 receptor coding sequences, several lists of different vertebrate species were proposed, classifying potential host susceptibility based on this data (Mathavarajah and Dellaire 2020). Combining both *in silico* and experimental data, SARS-CoV-2 susceptible animal hosts are now clearly defined: bats, felids, non-human primates, mustelids, and deer are highly susceptible (Islam et al. 2022; Palmer et al. 2021; Parolin et al. 2021); domestic dogs have low susceptibility, and other vertebrates such as sheep, birds, and reptiles are not susceptible (Villanueva-Saz et al. 2021; Fischhoff et al. 2021a). Besides mink farms, other places that have a high contact of human and wild animals are zoological parks and wildlife rehabilitation centers (WRCs, hereafter). Indeed, zoological parks were the first place where susceptible wild animals such as tigers and cougars were reported both with infection and clinical symptoms of SARS-CoV-2 (Jemeršić et al. 2021). Moreover, WRCs are places of high significance in their potential to spread SARS-CoV-2 infection to susceptible wildlife reservoir hosts; this due to the fact that animals that arrive at WRCs have several instances of interaction with humans: both with the people who find them, with the WRC trained staff that receives them, and later when they are released back to the environment (Hedman et al. 2021). Consequently, there are concerns that WRCs could act as a potential threat in the dissemination of SARS-CoV-2 from humans to wildlife (Islam et al. 2022; Sharun et al. 2021a), especially in places such as Latin America, where most WRCs are funded by visitors and donations, both heavily impacted by COVID-19 restrictions, and also because in Latin America there are many potentially susceptible wildlife species to SARS-CoV-2 infection (Chaves et al. 2021).

In Chile, the first reported human case of COVID-19 was on March 3rd, 2020, with the first wave between May and June 2020 with 231,948 reported cases, and the second wave between February and March 2021 with 470,542 reported cases. Vaccination started on December 24^th^, 2020 with both inactivated and mRNA vaccines. As of November 2022, the total accumulated number of confirmed cases in Chile is 4,769,638 with 50,063 deceased (MINSAL 2022). Regarding WRCs in Chile, there are currently 26 centers officially registered by the Chilean Agricultural and Livestock Service (SAG), and are distributed in several regions of the country (SAG 2022). Therefore, our aim was to perform molecular surveillance of SARS-CoV-2 at six WRCs and three wildlife associated institutions, located in different geographical areas, for viral detection in potentially susceptible native wild animals. To the best of our knowledge, there are no reports of molecular surveillance of SARS-CoV-2 in wildlife admitted at WRCs in Latin America and the rest of the world.

## 2. Materials and methods

The authors confirm that the ethical policies of the journal, as noted on the journal’s author guidelines page, have been adhered to and the appropriate ethical review committee approval has been received. This study was approved by the Universidad Andres Bello Ethics Board, protocol number 041/2020. This study was conducted for a year, between October 2020 and October 2021. The Chilean Agricultural and Livestock Service (SAG) authorized the WRCs to be able to work with wildlife with the following permits: N° 1506/2012, 803/2014, 1355/2015, 3717/2015, 132/2017, 455/2017, 2186/2019, 7490/2021.

### 2.1. Animal selection and sampling

The survey was conducted in six WRCs and three wildlife associated institutions (WRC1-WRC9). WRCs 1,3,4,6-8 received, treated and released/euthanized wildlife. WRC2 operated only as an exhibition center, WRC5 handled exclusively road-accidents, and WRC9 captured, sampled, and then released animals. Sampling kits were sent to each WRC. They contained personal protective equipment (PPE), disinfectants, and swabs with DNA/RNA shield (Zymo Research, Irvine, CA, USA). Each WRC received materials to sample at least 10 different animals. Wild mammals in Chile usually are admitted at WRCs after trauma (Romero et al. 2019), nonetheless, the common admittance causes for animals sampled in this study were mostly trauma, followed by disease and orphaning. Following the OIE Guidelines of Handling wild animals during the COVID-19 pandemic (OIE 2022), no animals were anesthetized solely for the purpose of obtaining a sample, and they were only sampled when other medical procedures had to be performed. Also, local veterinary staff determined that sampling the animals did not put them at risk in the current condition that they were admitted. Because of the aforementioned factors, there was no standardized number of sampled animals or time frame of sampling, as it was performed based on opportunity.

The is no national entity that regulates the measures WRCs should take to handle susceptible individuals during the pandemic, each WRC had their own protocol to try and not inadvertently infect the individuals. In most WRCs, personal protection equipment was used such as, KN95 or surgical masks, nitrile gloves, goggles or face shields, gowns or aprons, surgical caps and disposable shoe covers to handle all susceptible patients. Furthermore, before and after treating or working with the patients, surfaces were thoroughly disinfected with quaternary ammonium, to decrease the probability of having cross species infections between different sampled individuals. WRC staff were not regularly screened; however, if they become PCR positive to COVID-19 they could not come back to work unless 2 weeks had passed due to national legislative protocols that were in place during this study. Furthermore, if the staff are a close contact or confirmed case, they have an immediate medical license to stay at home until 2 weeks have passed.

Potentially infected animal selection criteria were as followed: 1. Confirmed species positivity reported in the literature; 2. Possible infection based on ACE2 receptor aminoacidic sequences; 3. Possible infection based on the taxonomic family of previously reported SARS-CoV-2 positive species.

### 2.2. Sample collection

In all live animals, one sample was obtained by two nasopharyngeal swabs in animals with large nostrils (one for each nostril) or one oropharyngeal swab in animals with nostrils smaller than the swab diameter. In necropsied animals, one tracheal swab was obtained. In almost all animals (live and necropsied), a second rectal swabbing was performed with one swab. All live sampling was performed in previously anesthetized animals by qualified veterinary staff in charge of each WRC. Each collection tube had 1 mL of DNA/RNA shield, which allows virus inactivation and RNA stabilization until extraction (Dunbar and Tang 2022). Swabs were then frozen at −20 °C until they were transported on dry ice to the laboratory. Samples from WRCs 1-6 and 8 were processed within a week of being obtained. Samples from WRCs 7 and 9 were stored frozen at −20 °C for a month and then shipped to the laboratory.

### 2.3. RNA isolation

Whole RNA was isolated from the samples using the Rneasy® Mini kit (QIAGEN, Germantown, MD, USA) with a maximum of 600 µL, following the manufacturer’s recommendations, with an elution volume of 50 µL. RNA was quantified by absorbance using a Qubit 4 Fluorometer, and only samples with >1 µg of total RNA were included in the study. An aliquot of 10 µL was separated for One-Step RT-qPCR assays and stored at −20 °C. The remaining 40 µL were immediately retrotranscribed to cDNA.

### 2.4. cDNA synthesis

RNA was retrotranscribed to cDNA using the Quantitec® reverse transcription kit (QIAGEN, USA). The manufacturer’s recommendations were as follows, with a step to eliminate contamination from genomic DNA (gDNA): 2 µL of gDNA Wipeout Buffer 7X, 2 µL of Rnase-free water and 10 µL of template RNA were incubated for 2 min at 42 °C. Afterwards, the template RNA was added to the reverse-transcription master mix and the incubation was carried out in one step: 30 min at 42 °C and 3 min at 95 °C. cDNA samples were stored at −20 °C until the following molecular analyses.

### 2.5. RT-PCR assays of Pan-Coronavirus (Pan-CoV)

A first screening was performed to evaluate the overall presence of Coronavirus in the samples, following the protocol described by Hu et al. (Hu et al. 2018), as previously validated for SARS-CoV-2 (Erlichster et al. 2021). The RT-PCR protocol was performed as follows: 10 µM of primers Pan-CoV-18 F2 (5′-AARTTYTAYGGHGGYTGG-3′) and Pan-CoV-18 R1 (5′-GARCARAATTCATGHGGDCC-3′), 5X Green GoTaq® Flexi Buffer (Fitchburg, WI, USA), 10 mM of dNTPs, MgCl_2_ solution 25 mM, GoTaq® G2 Flexi DNA Polymerase, nuclease free water and 2 µL of DNA template, in a final volume of 20 µL. Cycling conditions were 30 min at 50 °C, 2 min at 95 °C, followed by 35 cycles at 94 °C for 40 s, 52 °C for 40 s and 72 °C for 45 s, finishing with 72 °C for 5 min. A sample was considered positive with an amplicon of 668 bp. As a positive control we used a cDNA extracted from Nobilis IB MA5 vaccine and nuclease-free water as the no-template control in each assay.

### 2.6. Real-time PCR assays of SARS-CoV-2 (RT-qPCR)

qPCR assays were performed using the SARS-CoV-2 GenomeCoV19 Detection Kit (Applied Biological Materials Inc., Richmond, BC, Canada). The optimized protocol, ABM.G628V2-200M, consisted in COVID-19 Primers/Probes (G628-1.V2), RT-qPCR Enzyme mix (RT-13), Luna® Universal Probe qPCR master mix (Ipswich, MA, USA) and 6 μL of RNA template, in a final volume of 20 μL. Cycling conditions were 15 min at 50 °C, 2 min at 95 °C followed by 3 cycles at 95 °C for 5 s and 60 °C for 15 s, finishing with a 40 cycles at 90 °C for 5 s and 60 °C for 30 s. Detection of SARS-CoV-2 was considered positive when the two fluorophores FAM and HEX were amplified (N and S genes). All the assays were run in a Bioer LineGene k plus FQD-48A Real-time PCR (Hangzhou, Binjiang District, China), using the positive control template and negative extraction control included in the kit, and nuclease-free water as the no-template control in each assay.

## 3. Results

### 3.1. Susceptible native wild animals sampled

**Figure 1.**
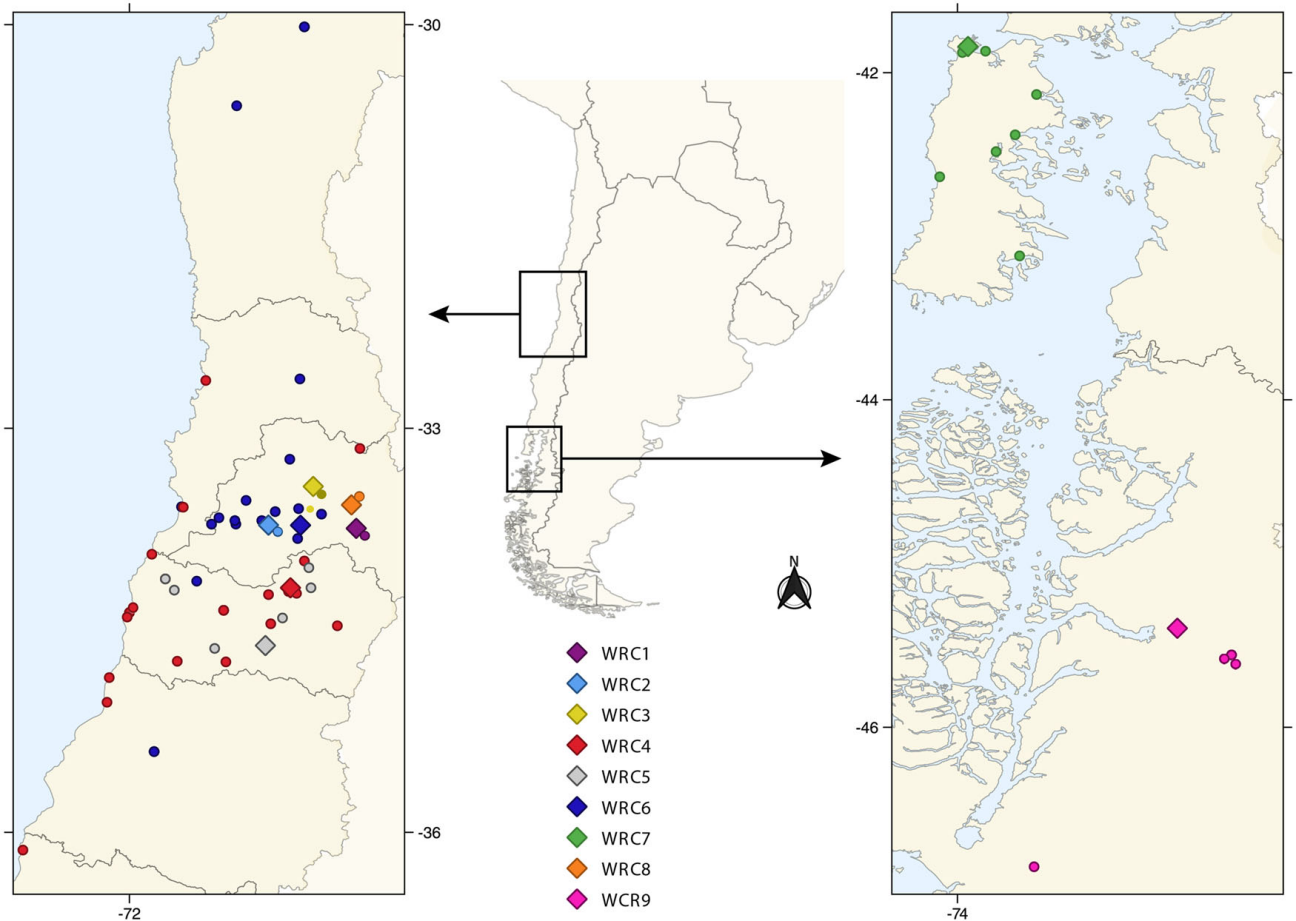
Geographical distribution of sampled animals. Each dot represents where the animal was found, and each diamond represents the location of Wildlife Rehabilitation Centers (WRC) where the animal was admitted for sampling (WRC1-WRC9). The color of each circle indicates the respective WRC where the animals were sampled.

**Figure 2.**
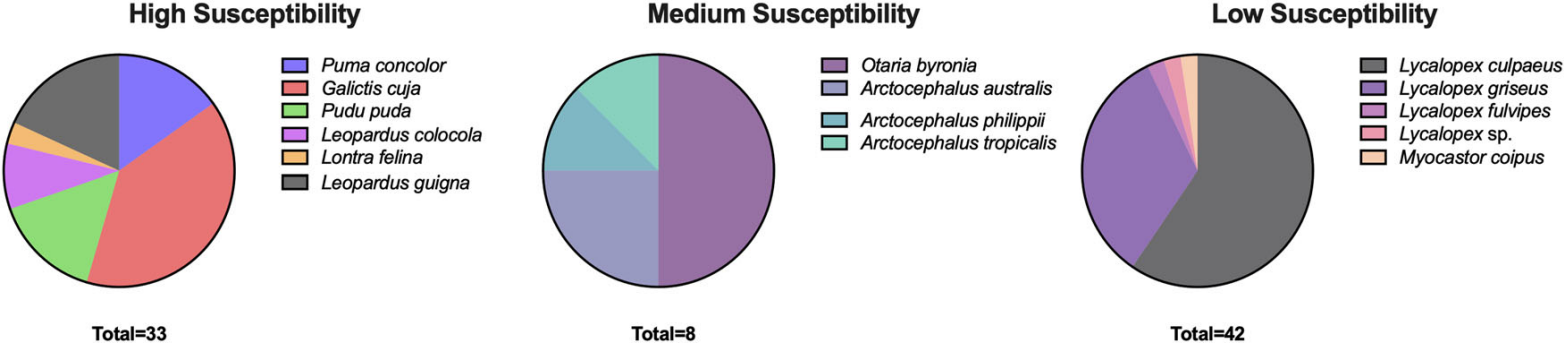
Susceptibility to SARS-CoV-2 infection of sampled animals based on published data (Islam et al. 2022; Palmer et al. 2021; Parolin et al. 2021; Villanueva-Saz et al. 2021; Fischhoff et al. 2021a; 2021b). Since there are no *in silico* or experimental susceptibility reports for most species (*Galictis cuja*, *Pudu puda*, *Lontra felina*, *Lycalopex culpaeus*, *Lycalopex griseus*, *Lycalopex fulvipes*, *Otaria byronia*, *Arctocephalus australis*, *Arctocephalus philippi*, *Arctocephalus tropicalis*, *Leopardus guigna*, and *Leopardus colocola*), susceptibility was estimated based on taxonomic family. Each color represents the proportion of sampled individuals in each susceptibility category.

A total of 185 samples were obtained from 83 individuals belonging to 15 different species in 9 WRCs (Figure 1). Species were classified into low, medium, and high susceptibility to SARS-CoV-2, according to available *in silico* and experimental infection data (Fischhoff et al. 2021a; Figure 2).

### 3.2. Pan-CoV RT-PCR results

All 185 samples were negative to the Pan-Cov RT-PCR assay.

### 3.3. SARS-CoV-2 qPCR assay results

All 185 samples were tested with two different qPCR assays, and all animals were negative to both. The number and type of samples analyzed per species are available in Table 1. Detailed results for each specimen, such as sex, cause of admission and type of sample assayed are available in Table 2.

**Table 1.** Total number of wild animals sampled in this study. Each sampled animal is identified with common name, scientific name, number of sampled animals, wildlife rehabilitation center, number and kind of sample analyzed, and qPCR assay results.

Common Name	Scientific Name	Nº of specimens	WRC	OS	NS	RS	TS	FAM qPCR	HEX qPCR
Mountain Lion	*Puma concolor*	5	WRC1, WRC2, WRC3	0	5	5	0	Negative	Negative
Lesser Grison	*Galictis cuja*	13	WRC3, WRC4, WRC5, WRC6	12	0	11	1	Negative	Negative
Pudu	*Pudu puda*	5	WRC7	0	5	5	0	Negative	Negative
Pampas Cat	*Leopardus colocola*	3	WRC3, WRC6	0	1	1	0	Negative	Negative
Kodkod	*Leopardus guigna*	6	WRC5, WRC7, WRC9	2	2	4	0	Negative	Negative
Marine Otter	*Lontra felina*	1	WRC4	0	0	1	0	Negative	Negative
South American Sea Lion	*Otaria byronia*	4	WRC4	4	0	3	0	Negative	Negative
South American Fur Seal	*Arctocephalus australis*	2	WRC4, WRC7	0	1	2	0	Negative	Negative
Subantarctic Fur Seal	*Arctocephalus tropicalis*	1	WRC4	0	1	0	0	Negative	Negative
Juan Fernández Fur Seal	*Arctocephalus philippii*	1	WRC4	1	0	1	0	Negative	Negative
Andean Fox	*Lycalopex culpaeus*	25	WRC1, WRC4, WRC6, WRC8	3	8	19	12	Negative	Negative
South American Gray Fox	*Lycalopex griseus*	14	WRC4, WRC6	2	4	12	5	Negative	Negative
Darwin’s Fox	*Lycalopex fulvipes*	1	WRC7	0	1	0	0	Negative	Negative
Fox	*Lycalopex* sp.	1	WRC4	1	0	1	0	Negative	Negative
Coypu	*Myocastor coipus*	1	WRC6	1	0	1	0	Negative	Negative

WRC = Wildlife rehabilitation center; OS = oropharyngeal swab; NS = nasal swab; RS = rectal swab; TS = tracheal swab; WRC2 operates as a wildlife exhibition center; WRC5 and WRC9 did wildlife sampling but not rehabilitation.

**Table 2.** Animal species sampled, sample kind, sex, cause of admission and wildlife rehabilitation center of admittance. Samples are listed in chronological order.

Species	Sample Kind	Sex	Cause of Admission	Wildlife Rehabilitation Center
*Puma concolor*	NS	Male	Orphaned	WRC1
	RS			
*Puma concolor*	NS	Male	Orphaned	WRC1
	RS			
*Lycalopex culpaeus*	NS	Female	Orphaned	WRC4
*Puma concolor*	NS	Female	Orphaned	WRC2
	RS			
*Galictis cuja*	OS	Male	Trauma	WRC4
	RS			
*Lycalopex culpaeus*	OS	Female	Trauma	WRC4
	RS			
*Otaria byronia*	OS	Male	Trauma	WRC4
	RS			
*Arctocephalus philippii*	OS	–	Orphaned	WRC4
	RS			
*Lycalopex culpaeus*	RS	Female	Trauma	WRC4
*Lycalopex griseus*	NS		Ilegal trapping	WRC6
	RS			
*Lycalopex culpaeus*	NS	Male	Collision with vehicle	WRC6
	RS			
*Lycalopex culpaeus*	NS	Male	Disease (Distemper)	WRC6
	RS			
*Lycalopex griseus*	TS	Female	Orphaned	WRC6
	RS			
*Lycalopex griseus*	TS	Female	Collision with vehicle	WRC6
	RS			
*Leopardus colocola*	NS	Male	Collision with vehicle	WRC6
	RS			
*Lycalopex griseus*	RS	Female	Ilegal captivity	WRC6
*Otaria byronia*	NS	Male	Trauma	WRC4
*Galictis cuja*	OS	Male	Collision with vehicle	WRC5
	RS			
*Lycalopex culpaeus*	NS	Male	Sarna	WRC4
*Lycalopex griseus*	NS	Male	Disease (Distemper)	WRC4
*Galictis cuja*	OS		Collision with vehicle	WRC4
	RS			
*Galictis cuja*	OS		Collision with vehicle	WRC4
	RS			
*Lycalopex* sp.	OS		Orphaned	WRC4
	RS			
*Galictis cuja*	OS		Collision with vehicle	WRC4
	RS			
*Galictis cuja*	OS	Female	Collision with vehicle	WRC5
	RS			
*Leopardus guigna*	OS		Orphaned	WRC5
	RS			
*Otaria byronia*	OS		Orphaned	WRC4
	RS			
*Galictis cuja*	OS	Male	Collision with vehicle	WRC4
	RS			
*Lycalopex culpaeus*	TS	Male	Unknown/Not Recorded	WRC8
	RS			
*Lycalopex culpaeus*	OS		Unknown/Not Recorded	WRC1
	RS			
*Lycalopex culpaeus*	TS		Collision with vehicle	WRC6
*Lycalopex culpaeus*	TS	Male	Unknown/Not Recorded	WRC8
	RS			
*Lycalopex culpaeus*	NS		Unknown/Not Recorded	WRC1
	RS			
*Lycalopex culpaeus*	NS		Unknown/Not Recorded	WRC1
	RS			
*Lycalopex culpaeus*	TS	Female	Unknown/Not Recorded	WRC8
	RS			
*Lycalopex culpaeus*	TS	Male	Unknown/Not Recorded	WRC8
	RS			
*Lycalopex culpaeus*	TS		Juvenile with suboptimal condition	WRC6
	RS			
*Lycalopex culpaeus*	TS		Attacked by dog	WRC6
	RS			
*Galictis cuja*	OS	Female	Trauma	WRC4
	RS			
*Galictis cuja*	OS	–	Dead	WRC4
	RS			
*Lycalopex griseus*	RS	Female	Disease (Scabies)	WRC4
*Lycalopex griseus*	OS	Female	Trauma	WRC4
	RS			
*Otaria byronia*	OS	Female	Orphaned	WRC4
	RS			
*Galictis cuja*	OS	Female	Trauma	WRC4
	RS			
*Lycalopex griseus*	NS	Female	Disease (Scabies)	WRC4
*Galictis cuja*	OS	Male	Malnutrition	WRC4
*Galictis cuja*	OS		Unknown/Not Recorded	WRC5
*Puma concolor*	NS		Unknown/Not Recorded	WRC3
	RS			
*Puma concolor*	NS	Female	Unknown/Not Recorded	WRC3
	RS			
*Galictis cuja*	TS	Male	Collision with vehicle	WRC6
	RS			
*Leopardus colocola*		Female	Unknown/Not Recorded	WRC3
*Leopardus colocola*		Female	Unknown/Not Recorded	WRC3
*Myocastor coipus*	OS	Male	Unknown trauma	WRC6
	RS			
*Lontra felina*	RS	Male	Trauma	WRC4
*Arctocephalus tropicalis*	NS	–	Orphaned	WRC4
	RS			
*Lycalopex fulvipes*	NS		Unknown/Not Recorded	WRC7
*Pudu puda*	NS		Unknown/Not Recorded	WRC7
	RS			
*Pudu puda*	NS	Female	Unknown/Not Recorded	WRC7
	RS			
*Leopardus guigna*	NS	Male	Unknown/Not Recorded	WRC7
	RS			
*Pudu puda*	NS	Female	Unknown/Not Recorded	WRC7
	RS			
*Pudu puda*	NS	Female	Unknown/Not Recorded	WRC7
	RS			
*Arctocephalus australis*	NS		Unknown/Not Recorded	WRC7
	RS			
*Arctocephalus australis*	NS	Male	Trauma	WRC4
	RS			
*Lycalopex culpaeus*	TS		Unknown trauma	WRC6
	RS			
*Lycalopex culpaeus*	TS		Poisoned	WRC6
	RS			
*Lycalopex griseus*	TS		Attacked by dog	WRC6
	RS			
*Lycalopex griseus*	RS		Collision with vehicle	WRC6
	TS			
*Lycalopex griseus*	TS		Ilegal trapping	WRC6
	RS			
*Lycalopex griseus*	OS		Orphaned	WRC6
	RS			
*Lycalopex culpaeus*	TS		Juvenile with suboptimal condition	WRC6
	RS			
*Lycalopex culpaeus*	OS		Unknown/Not Recorded	WRC4
	RS			
*Lycalopex culpaeus*	NS	Female	Disease (Gut Infection)	WRC4
	RS			
*Lycalopex culpaeus*	OS	Female	Trauma	WRC4
	RS			
*Lycalopex griseus*	NS	Female	Trauma	WRC4
	RS			
*Lycalopex griseus*	RS		Unknown/Not Recorded	WRC4
*Otaria byronia*	OS	Female	Unknown/Not Recorded	WRC4
	RS			
*Lycalopex culpaeus*	OS	Male	Trauma	WRC4
	RS			
*Lycalopex culpaeus*	NS	Female	Unknown/Not Recorded	WRC5
	RS			
*Leopardus guigna*	NS		Unknown/Not Recorded	WRC9
*Leopardus guigna*	RS		Unknown/Not Pecorded	WRC9
*Leopardus guigna*	OS		Unknown/Not Recorded	WRC9
*Leopardus guigna*	RS		Unknown/Not Recorded	WRC9

WRC = Wildlife rehabilitation center; OS = oropharyngeal swab; NS = nasal swab; RS = rectal swab; TS = tracheal swab; WRC2 operates as a wildlife exhibition center; WRC5 and WRC9 did wildlife sampling but not rehabilitation.

## 4. Discussion

Most of the evaluated animals were sampled within the first days of admission during their initial physical exams, and due to their negative results, this could be indicating that these individuals are not getting infected in their previous natural habitat. This is in accordance with previous studies reporting that the evidence of the maintenance of the virus in the wild is scant (Delahay et al. 2021), although the exposure of wild animals to the virus has been reported (Chandler et al. 2021). Preventive measures adopted at WRCs will continue to be followed when necessary, as they could prevent transmission from asymptomatic staff. However, the cross-sectional design of our study prevented more permanent monitoring of the animals, which were only sampled when they were subjected to other interventions that required their direct manipulation. In addition, serological survey of antibodies against SARS-CoV-2 should be included in future surveillance and monitoring, to obtain information of virus exposure in wildlife. Ideally, future studies should also monitor the WRC personnel SARS-CoV-2 infection status in a periodic manner, to test the animals in case there are human cases of COVID-19 in the compound, which is a scenario that did not happen during this study. Asymptomatic healthcare workers have been identified as critical points in SARS-CoV-2 transmission, as they cannot do physical distancing from patients. In this group, the use of PPE has been paramount in preventing SARS-CoV-2 transmission (Olmos et al. 2021). This also applies to WRCs staff, which is why the use of face masks, face shields, gloves, disposable overalls, and shoe covers is mandatory in most of the WRCs included in this study.

Vaccination for preventing SARS-CoV-2 is not yet performed in animals from these WRCs, so this would not be influencing our results. Also, a large proportion of the samples analyzed in this study were obtained when vaccination was not yet available to neither domestic and wild animals, the general public, animal handlers, nor the veterinarians in all the WRCs included in this study. Currently, in Chile 92.1% of the population is fully vaccinated (either with a single or two shots), and 78.3% has received a booster immunization (MINSAL 2022). This vaccination effort is likely to contribute to a smaller chance of horizontal viral transmission between humans and wildlife. Now, vaccination of both domestic and wild animals is a possibility, since vaccine candidates were first tested in non-human animals prior to clinical trials; domestic cats have shown high levels of neutralizing antibodies, and non-human primates in zoos have also been immunized (Khan Sharun et al. 2021b). However, vaccination at WRCs is not warranted, because these vaccination efforts are unlikely to prevent SARS-CoV-2 infection in wild animals, therefore, vaccinating wild animals is not a part of the animal handling protocols at WRCs, and they usually can only receive one shot. Nonetheless, domestic animals and captive animals at zoos should be vaccinated to minimize symptoms and risk of severe disease.

According to the International Union for Conservation of Nature (IUCN), some animals sampled in this study belong to vulnerable (*Pudu puda*, *Leopardus guigna*), near threatened (*Leopardus colocola*), and endangered (*Lontra felina*, *Lycalopex fulvipes*) categories. Since felids, mustelids and cervids have been reported previously naturally infected by SARS-COV-2, the possibility of SARS-CoV-2 infection was of high concern, enhancing the need to assess the infection in those mentioned species. Regarding the other taxonomic groups, negative results were previously reported in wild canids (Jemeršić et al. 2021), and, to the best of our knowledge, this is the first assessment of the infection in Otariidae and Myocastoriidae. We are confident in our results, because the RT-PCR and RT-qPCR assays have high sensitivity and specificity for SARS-CoV-2 infection (Camporesi et al. 2022; Pratelli et al. 2022), the GenomeCoV19 Detection Kit used in this study has been validated previously (Buchta et al. 2021; Wozniak A et al. 2020; Peña et al. 2021; Sarwar et al. 2021); with only one of these publications reporting a 95% sensitivity and 100% specificity (Wozniak A et al. 2020). This commercial kit is designed with two different SARS-CoV-2 genes (N and S), minimizing the possibility of false positives. To further prove our results, each of these animals were evaluated from two different biological samples. However, since the OIE guidelines recommended not to sample animals at zoos and WRCs for the sole purpose of detecting SARS-CoV-2 (OIE 2022), animals were sampled once and only during other procedures, which could lead to false negatives (Sánchez‐Montes et al. 2022).

The main limitation of our study lies in the sampling method; because of the limited manipulation that each animal was subjected to, we could only sample each individual once. Also, due to anatomical differences, the sample kind also fluctuated, as we were unable to obtain nasal swabs from every species, since the nostrils of small carnivores were narrower than the swabs used. Future efforts to monitor infected wildlife that are admitted at WRCs and released back to the environment should include at least 2 samplings, one at admittance and the other before release. In this way we could ensure that released fauna are not carrying SARS-CoV-2 infection back to nature.

## 5. Conclusion

Our study constitutes the first report on the molecular surveillance of SARS-CoV-2 from wildlife treated in rehabilitation centers of Chile. Efforts must be made to continue molecular surveillance of SARS-CoV-2, especially in cervids, and serological assays must be implemented to assess previous exposure to the virus. Both assays should be performed in released fauna, to ensure ecosystem and planetary health.
